# Access to Transplantation and Transplant Outcome Measures (ATTOM): study protocol of a UK wide, in-depth, prospective cohort analysis

**DOI:** 10.1136/bmjopen-2015-010377

**Published:** 2016-02-25

**Authors:** Gabriel C Oniscu, Rommel Ravanan, Diana Wu, Andrea Gibbons, Bernadette Li, Charles Tomson, John L Forsythe, Clare Bradley, John Cairns, Christopher Dudley, Christopher J E Watson, Eleanor M Bolton, Heather Draper, Matthew Robb, Lisa Bradbury, Rishi Pruthi, Wendy Metcalfe, Damian Fogarty, Paul Roderick, J Andrew Bradley

**Affiliations:** 1 Transplant Unit, Royal Infirmary of Edinburgh, Edinburgh, UK; 2 Richard Bright Renal Unit, Southmead Hospital, Bristol, UK; 3 Health Psychology Research Unit, Royal Holloway, University of London, London, UK; 4 Department of Health Services Research and Policy, London School of Hygiene and Tropical Medicine, London, UK; 5 Department of Renal Medicine, Freeman Hospital, Newcastle upon Tyne, UK; 6 Department of Surgery, University of Cambridge and the NIHR Cambridge Biomedical Research Centre, Cambridge, UK; 7 School of Health and Population Sciences, University of Birmingham, Birmingham, UK; 8 NHS Blood and Transplant, Bristol, UK; 9 UK Renal Registry, Bristol, UK; 10 Primary Care and Population Sciences, Faculty of Medicine, University of Southampton, Southampton, UK

**Keywords:** TRANSPLANT SURGERY, QUALITATIVE RESEARCH, HEALTH ECONOMICS, EPIDEMIOLOGY

## Abstract

**Introduction:**

There is significant intercentre variability in access to renal transplantation in the UK due to poorly understood factors. The overarching aims of this study are to improve equity of access to kidney and kidney–pancreas transplantation across the UK and to optimise organ allocation to maximise the benefit and cost-effectiveness of transplantation.

**Methods and analysis:**

6844 patients aged 18–75 years starting dialysis and/or receiving a transplant together with matched patients active on the transplant list from all 72 UK renal units were recruited between November 2011 and March 2013 and will be followed for at least 3 years. The outcomes of interest include patient survival, access to the transplant list, receipt of a transplant, patient-reported outcome measures (PROMs) including quality of life, treatment satisfaction, well-being and health status on different forms of renal replacement therapy. Sociodemographic and clinical data were prospectively collected from case notes and from interviews with patients and local clinical teams. Qualitative process exploration with clinical staff will help identify unit-specific factors that influence access to renal transplantation. A health economic analysis will explore costs and outcomes associated with alternative approaches to organ allocation. The study will deliver: (1) an understanding of patient and unit-specific factors influencing access to renal transplantation in the UK, informing potential changes to practices and policies to optimise outcomes and reduce intercentre variability; (2) a patient-survival probability model to standardise access to the renal transplant list and (3) an understanding of PROMs and health economic impact of kidney and kidney–pancreas transplantation to inform the development of a more sophisticated and fairer organ allocation algorithm.

**Ethics and dissemination:**

The protocol has been independently peer reviewed by National Institute for Health Research (NIHR) and approved by the East of England Research Ethics Committee. The results will be published in peer-reviewed journals and presented at conferences.

**Strengths and limitations of this study**

- First research programme involving all renal and transplant units in the UK.
- An in-depth analysis (quantitative and qualitative) of access to transplantation and transplant outcome.
- Correlation with patient-reported outcome measures, health status and quality of life.
- Health economic analysis exploring costs and outcomes associated with alternative approaches to organ allocation.
- Limitation due to recruitment process and comorbidity data recorded at enrolment rather than same time point for all study cohorts.

## Introduction

Kidney transplantation is widely regarded to be the best treatment for selected patients with end-stage renal disease (ESRD). When compared with dialysis, transplantation leads to a twofold to threefold increase in life expectancy and, it is often believed, a better quality of life (QoL).^1–4^ Over the last decade, transplant survival results have improved progressively and 1-year, 5-year and 10-year graft survival rates are now >90%, >70% and >60%, respectively. For selected patients with ESRD due to type 1 diabetes, combined (or simultaneous) pancreas and kidney (SPK) transplantation offers a better life expectancy compared with renal transplantation alone (70% vs 30% at 10 years^5^ and ameliorates diabetes complications).^5^ ^6^

These successes have led to a greater demand for transplantation with an ever increasing gap between supply and demand. The demography of patients with ESRD is also changing with an ageing population having more comorbid conditions that may preclude transplantation.^7^ ^8^ Currently, fewer than 40% of all patients with ESRD in the UK are listed as suitable candidates for transplantation and only carefully selected patients, without severe cardiovascular disease, undergo an SPK transplant. The need for research on the impact of pretransplant comorbidity on transplant outcome has been identified as a major priority in the UK by the Renal Association.^9^

It is important, in the interest of fairness and equity, that access to the transplant waiting-list is, so far as is possible, standardised, transparent and based on validated criteria. Recent evidence shows that access to transplantation varies between and within the UK centres and differences in assessment for comorbidity are likely to be a major reason.^10^ However, even when the effects of comorbidity are accounted for, there remains variation in access to transplantation suggesting that other centre-specific factors are implicated.^11–13^ It is unclear which patient-specific and centre-specific factors are responsible for such variations,^11^ ^14^ or indeed which centre practices represent the optimal approach. It is also unclear which patient-specific and centre-specific factors impact on outcomes following transplantation but the development of a standardised approach would enable an evidence-based decision-making at individual patient level.

Successful kidney transplantation appears to improve QoL and health status compared with dialysis, but the benefit may not be apparent in all patient groups^15–17^ and is not supported by all studies.^18^ Furthermore, the impact of kidney–pancreas transplantation on QoL has not been conclusively established.^19^ There is a growing body of evidence supporting the cost-effectiveness of transplantation,^20^ ^21^ but there are unresolved questions about which patients may benefit the most from transplantation and how organ allocation can be further optimised given scarce supply.

There is considerable interest in the development of organ allocation schemes based on net transplant benefit and significant work has already been undertaken in the context of liver transplantation^22^ and cardiothoracic transplantation^23^ in the UK and the USA. However, existing kidney allocation policies don't take into account the potential impact of comorbid disease on transplant outcome nor do they address the best use of the increasing number of extended criteria deceased donor organs.^24–28^ Recent research has quantified the benefit of kidney and SPK transplantation in order to develop a survival probability model as a basis for listing for transplantation (in the UK)^29^ or as a potential allocation model (in the USA).^30^ No work has yet been carried out incorporating cost-effectiveness, health status, QoL and other patient-reported outcome measures (PROMs) in any allocation algorithms.

In order to address some of these challenges in transplantation, the UK National Institute for Health Research (NIHR) Access to Transplantation and Transplant Outcome Measures (ATTOM) research programme has been developed by a consortium involving all renal and transplant units in the UK. The overarching aims of the programme are to investigate how we might maximise the net benefit to society from kidney and SPK transplantation, by selecting recipients in a robust and transparent way so as to achieve the best balance between cost, prolongation of life, QoL and acceptability to patients and wider society. The five related research aims of the study are listed below.

1. To identify patient-specific and centre-specific factors that influence (a) access to the transplant waiting-list and to develop a survival probability model as a basis for standardising access to the transplant waiting-list and (b) access to transplantation (deceased donor kidney and pancreas and living donor kidney) for wait-listed patients.
2. To identify patient-specific and centre-specific factors that influence patient survival for transplant wait-listed dialysis patients, after deceased donor kidney transplantation, after SPK transplantation, after living donor kidney transplantation and after pre-emptive transplantation (transplantation as a first mode of renal replacement therapy (RRT) prior to the initiation of dialysis treatment).
3. To evaluate QoL and other PROMs for patients on dialysis, after deceased donor kidney transplantation, after SPK transplantation, after living donor kidney transplantation, after pre-emptive transplantation, in waiting-list controls for kidney and SPK transplantation and in those whose transplants have failed following recruitment to ATTOM.
4. To perform a health economic analysis to explore costs and outcomes associated with alternative approaches to organ allocation.
5. To utilise survival, health status, QoL, treatment satisfaction and costs to determine an optimal organ allocation policy as defined by the maximisation of clinical and cost–benefits derived from transplantation.

We describe the study population and the methodology underpinning the study analyses.

## Methods and analysis

### Study population

**Figure 1:**
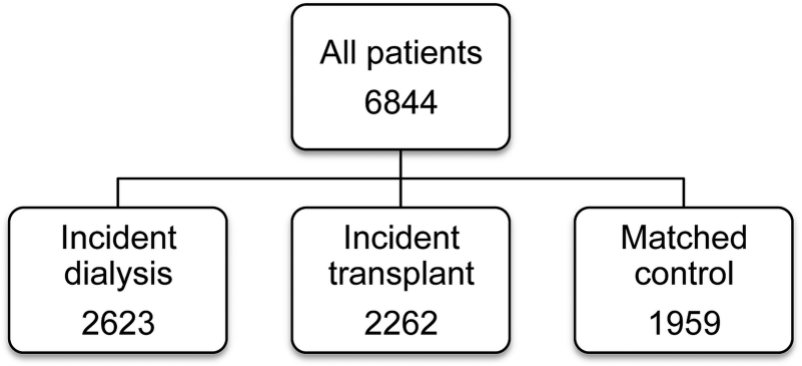
ATTOM, Access to Transplantation and Transplant Outcome Measures (ATTOM) study patient recruitment and cohort distribution.

**Figure 2:**
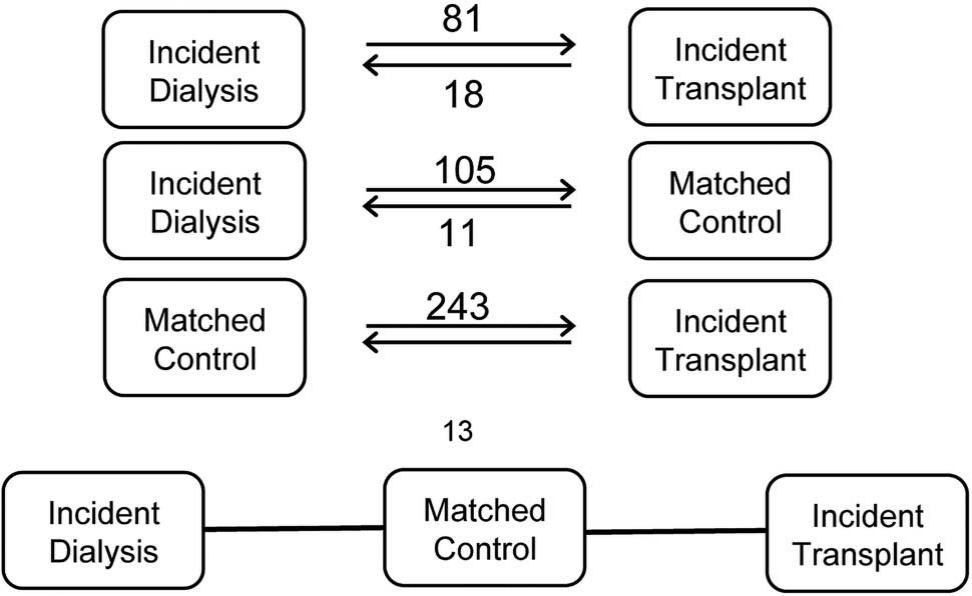
Number of patients changing between the study cohorts and the direction of change.

All 72 renal units (of which 23 are renal transplant units) in the UK contributed to the ATTOM programme. Between 1 November 2011 and 31 March 2013, 6360 patients aged 18–75 years were recruited in three cohorts: incident dialysis patients, incident kidney and SPK transplant patients and prevalent listed patients selected as controls for transplanted patients (figure 1). A total of 484 patients moved cohorts (13 patients moved twice) resulting in 6844 registrations within ATTOM (figure 2). In each centre, recruitment took place over a 1-year period aiming to include every patient <75 years of age starting RRT. Controls were selected automatically from the UK Transplant Registry database on a fortnightly basis and were matched for: age (within 5 years), time on the list, pre-emptive/on dialysis and the type of transplant (deceased donor or living donor).

**Figure 3:**
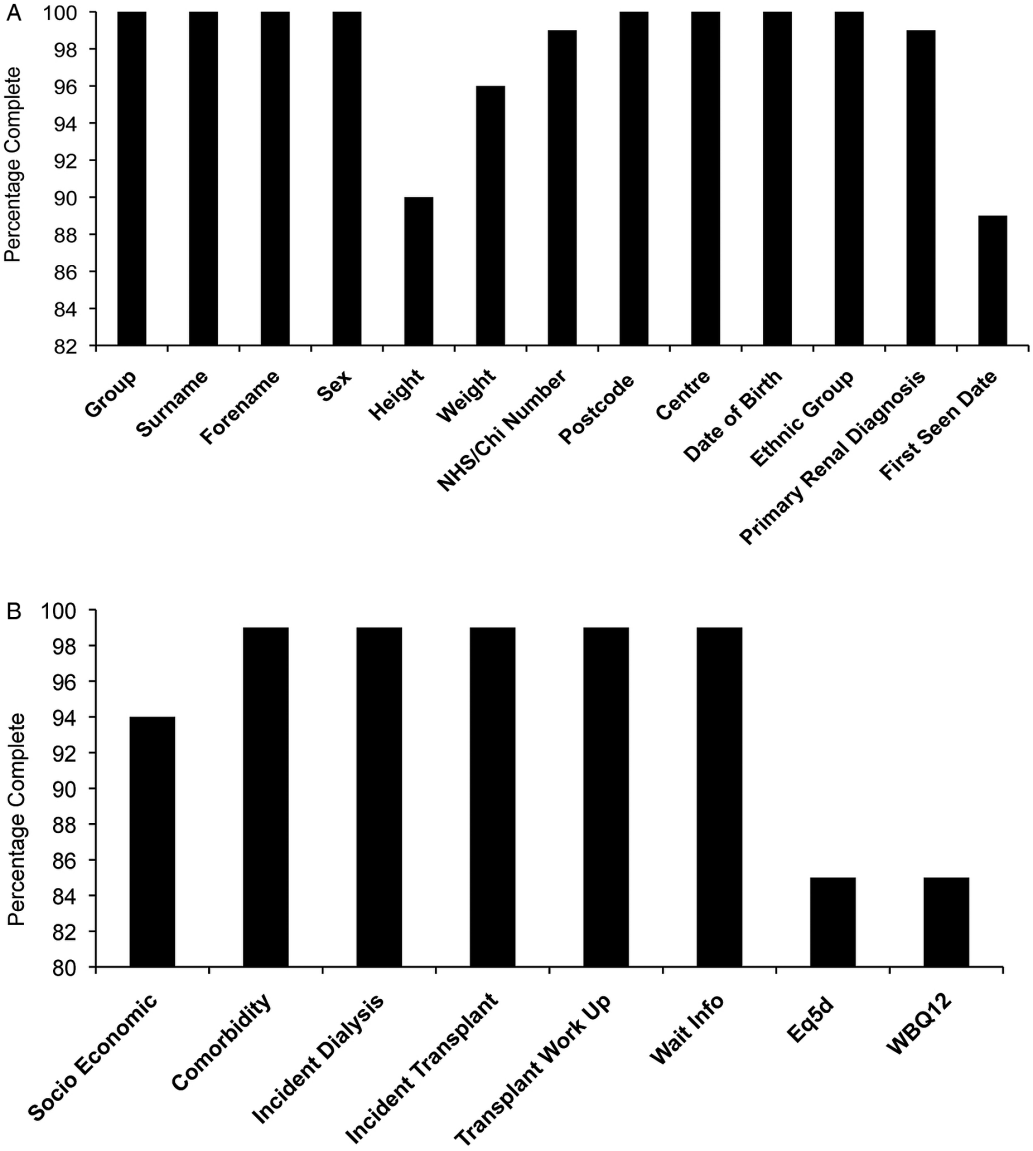
(A and B) Data completeness for each item collected in the study.

Patient-level data (see online supplementary appendix 1) were collected prospectively at the time of starting dialysis, at the time of transplantation or when identified as a control from the transplant list. Dedicated research nurses collected clinical and demographic information from the case notes and local electronic databases, and collected health status and well-being data from patients via completion of the EuroQoL five dimensions (EQ-5D)^31^ and 12-item Well-being Questionnaire (W-BQ12).^32–35^ The data were uploaded onto a secure website designed, developed and maintained by the UK Renal Registry (UKRR). Data completeness for the items recorded is illustrated in figure 3A, B. Data collection accuracy was ensured using uniform definitions and a training process for the research nurses. An independent data validation of coding of 5% of case notes in all research sites confirmed >98% concordance for all coded fields.

10.1136/bmjopen-2015-010377.supp1 Supplementary data

The demographic characteristics of the three study cohorts are illustrated in table 1.

**Table 1:** Demographic characteristics of the study cohorts

	Incident dialysis	Incident transplant	Matched controls
N	2623	2262	1959
Age at registration to ATTOM			
Mean±SD	56.18±13.55	49.34±13.44	50.38±12.83
Median (IQR)	58.39 (47.48–67.14)	50.28 (40.07–59.89)	51.14 (41.67–60.34)
Gender (%)			
Male	64.93	62.81	57.91
Female	35.07	37.19	42.09
Ethnicity (%)			
White	79.95	82.45	74.54
Asian	11.23	9.40	12.42
Black	7.09	6.21	10.93
Chinese	0.69	0.75	0.92
Mixed	0.65	0.80	0.87
Not specified	0.38	0.40	0.31
Age first seen by nephrologist			
Mean±SD	50.14±15.66	39.85±15.36	39.38±15.41
Median (IQR)	52.76 (39.85–62.68)	40.59 (28.65–51.61)	39.91 (28.24–51.48)

ATTOM, Access to Transplantation and Transplant Outcome Measures.

### Analysis

#### Access to transplantation

**Figure 4:**
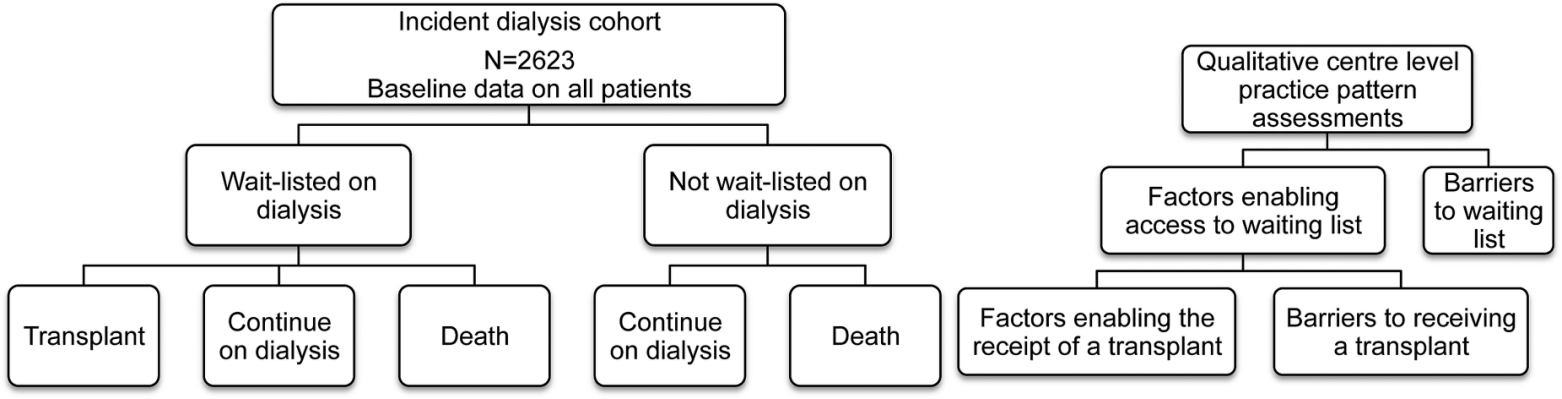
Quantitative and qualitative analysis approach for access to transplantation workstream.

Patient-level and centre-level factors influencing access to transplantation for patients starting dialysis are identified through quantitative and qualitative analysis. Patients are followed up for 4 years with data provided by the UKRR/Scottish Renal Registry and the UK Transplant Registry at National Health Service Blood and Transplant (NHSBT) in order to identify whether they are wait-listed for transplant or not, and if wait-listed, whether they received a transplant or not (figure 4). This will inform the analysis of the factors influencing access to listing after starting dialysis and subsequent transplantation.

The qualitative analysis aims to identify systems and processes consistently associated with better (or worse) outcomes in units across the UK, to help define best practice in transplant work-up and listing. This workstream consists of 40 initial qualitative interviews with key stakeholders and patients in a sample of 9 units stratified by proportion of listed dialysis patients, whether transplant or dialysis centre and geography to include spread of deprivation and ethnicity of the catchment area. This is followed by a purpose-designed structured questionnaire for use in a survey of all the UK renal and transplant units. A Delphi consensus study will provide better understanding of professional views on what characterises patients who should (and should not) be assessed for transplant listing and how they should be assessed. The Delphi study, undertaken by emailed electronic questionnaire with two rounds includes transplant surgeons and nephrologists from each centre. Participants are asked to agree or disagree with a series of statements about the eligibility criteria for listing. The initial overall responses are fed back and participants invited to reconsider their views in this second round prior to summarising final levels of agreement. Finally, both patient-level and centre-level factors (from the survey) are explored to determine their influence on transplant listing and subsequent access to transplantation.

#### Survival with transplantation versus dialysis

**Figure 5:**
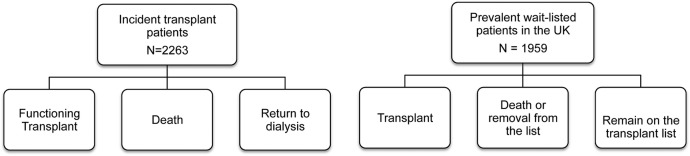
Study cohorts for survival analysis comparison.

Using data derived from the access to transplantation analysis, a multivariate Cox proportional hazards model will estimate the potential risk factors for mortality while on dialysis and their associated HRs, taking into account patient-level and centre-level factors in a multilevel modelling approach. Changes over time in the impact of factors measured at baseline on outcome are modelled using time-varying coefficients. Interactions between variables (eg, age and comorbidity) are included in the final model if significant. This will allow the development of a survival probability prediction tool, which can inform nationally agreed thresholds (such as ‘predicted survival >80% at 2 years after start of dialysis’) at which a patient should be activated and deactivated on the transplant list. The survival probability tool could be incorporated on a desktop or web-based platform enabling clinicians to discuss risk versus benefits with patients when considering transplant listing. A nationally agreed survival probability threshold will also enable robust intercentre comparison to audit listing practices. Follow-up of the dialysis cohort in conjunction with the cohorts illustrated in figure 5, beyond the 5-year duration of this project will enable further refinement of the survival probability assessment tool including the option to predict quality-adjusted life years gained with transplantation.

The study cohorts enable the analysis of patient-specific factors that influence survival for listed patients, after kidney transplantation (live and deceased donors) or after SPK transplantation. A multilevel modelling approach is used to analyse transplantation outcome data and the modelling explores how the outcome variables depend on one or more of the explanatory factors (patient and centre level). The models are developed on the basis of manual variable selection based on clinical and statistical input and are built up by repeatedly incorporating the most statistically significant variable and retesting all others in the presence of included variables, using clinical input to ensure development of a clinically appropriate model. Clinically relevant interactions between variables are predetermined and considered in the model building.

#### Evaluation of PROMs

All patients in the ATTOM programme were asked by the research nurses to complete measures of health status (using the EQ-5D and W-BQ12) at or soon after recruitment and at 6 months in those transplanted patients and matched controls on the waiting-list for transplant who were recruited during the first 6 months of nurse data collection. The EQ-5D provides an overall measure of perceived health ‘today’ and five individual items measuring mobility, pain, self-care, usual activities and anxiety/depression.^31^ The W-BQ12 has subscales to measure negative well-being (including depressed and anxious mood), energy and positive well-being over the past few weeks and an overall measure of general well-being.^32–35^ In addition, a detailed PROMs study on a subset of 652 ATTOM patients (table 2) recruited in a quasi-random manner (the first eligible patient for each group seen each month by each nurse) is evaluating QoL and the impact of the renal condition on QoL. This uses the individualised Renal-Dependent QoL (RDQoL) measure^36^ together with the Audit of Diabetes-Dependent QoL (ADDQoL) for people who also have diabetes^37^ ^38^ or a version of the ADDQoL with minor adaptations for people receiving an SPK transplant. These questionnaires are administered at 3 and 12 months post-transplant and at comparable times for those on dialysis. The Renal Treatment Satisfaction Questionnaire status (RTSQs) version^39^ is given alongside the RDQoL at each time point, and the Diabetes Treatment Satisfaction Questionnaire status (DTSQs^40^ ^41^) version is given to all those with diabetes (with minor adaptations for those who have received an SPK transplant). In addition, change versions of the RTSQ and DTSQ (the RTSQc and DTSQc)^42–44^ are given at 12 months to provide a direct comparison between satisfaction with current treatment and satisfaction with the treatment used before the study began. The EQ-5D and W-BQ12 are also included with the 12-month questionnaires in the detailed PROMs cohorts. The target patient groups and the timing of each questionnaire are summarised in table 3. Transplant recipients completed baseline questionnaires before transplantation where possible (patients receiving pre-emptive transplants) and within a few weeks of transplantation (deceased donor transplants). Patients were given the option to complete the questionnaires via telephone interviews or using mailed paper questionnaires.

**Table 2:** Detailed PROMs study group

Subgroup	Number of patients
Incident dialysis patients	147
Kidney transplant waiting-list patients	135
SPK transplant waiting-list	29
Deceased donor kidney transplant recipients	120
Living donor kidney transplant recipients	104
SPK transplant recipients	103
Failed transplant	14

PROMs, patient-reported outcome measures; SPK, simultaneous pancreas and kidney.

**Table 3:** Tools for QoL and other PROMs analysis, target population and timing of administration

Tool	Time of administration	Patient cohort
EuroQoL five dimensions (EQ-5D) health status tool	Recruitment 6 months 1 year*	All cohorts Those in first 6 months of data collection for transplant and matched control patients Patients in detailed PROMs cohort
Well-Being Questionnaire (W-BQ12)	Recruitment 6 months 1 year*	All cohorts Those in first 6 months of data collection for transplant and matched control patients Patients in detailed PROMs cohort
Renal-Dependent Quality of Life (RDQoL) Questionnaire	3 months* 1 year*	Patients in detailed PROMs cohort
Renal Treatment Satisfaction Questionnaire—status version (RTSQs)	3 months* 1 year*	Patients in detailed PROMs cohort
Renal Treatment Satisfaction Questionnaire—change version (RTSQc)	1 year*	Patients in detailed PROMs cohort
Audit of Diabetes-Dependent Quality of Life (ADDQoL) Questionnaire†	3 months* 1 year*	Patients in detailed PROMs cohort who have diabetes
Diabetes Treatment Satisfaction Questionnaire—status version (DTSQs)†	3 months* 1 year*	Patients in detailed PROMs cohort who have diabetes
Diabetes Treatment Satisfaction Questionnaire—change version (DTSQc)†	1 year*	Patients in detailed PROMs cohort who have diabetes

*Detailed PROMs cohort only.

†Modified versions of these questionnaires were completed by recipients of deceased donor SPK transplants.

PROMs, patient-reported outcome measures; QoL, quality of life; SPK, simultaneous pancreas and kidney.

Demographic and clinical data are used by the health psychologists alongside QoL and PROMs using multilevel modelling techniques in investigating the factors determining QoL measured by the RDQoL and health status measured by the EQ-5D and exploring the relationship between these two outcomes.

Sixty of the detailed PROMs patients (including patients from each treatment group purposively sampled to include those reporting above and below the mean for their treatment group on RDQoL scores) are included in a qualitative interview study to elicit further information about their experiences, with particular interest in variations in QoL, reasons for satisfaction or dissatisfaction with treatment and their understanding and views about the current and future possible organ allocation schemes.

#### Health economic analysis

The proposed health economic analysis focuses on the development of a model to simulate different approaches for allocating deceased donor kidneys to patients on the transplant waiting-list. Rather than attempting to identify one optimal allocation scheme, the analysis explores a range of conceptual schemes that reflect varying levels of emphasis on the principles of equity and efficiency. Each allocation scheme is evaluated in terms of cost and health outcomes captured by estimating quality-adjusted life years (QALYs).

The model is developed as a discrete event simulation (DES). This approach offers the flexibility to incorporate the influence of patient-level characteristics, such as age and comorbidities, in the estimation of both costs and health gains, to model competing risks and to capture the dynamic consequences of the allocation process for all patients subject to a constrained supply of donor organs.^45^ The model is populated using various sources of data with costs of RRT from NHS reference costs and variable hospital costs drawn on patient-level resource use from Hospital Episodes Statistics (HES). Survival for patients on the waiting-list and following transplant is estimated by fitting predictive models to historical data from NHSBT, while health state utility estimates are based on EQ-5D data prospectively collected in the ATTOM study.

#### Novel allocation schemes

An important outcome of ATTOM is to propose alternative organ allocation policies that consider efficiency and equity factors as well as QoL gains from transplantation utilising data on survival, health status, QoL and financial costs.

Under the current UK allocation scheme, kidneys are allocated according to an algorithm that among other variables favours those who have waited longest and have a better tissue-type match to the donated organ. Apart from avoiding extreme age mismatches, no account is taken of other more complex indicators such as the ‘quality’ of the kidney, patient QoL and cost-effectiveness of different types of transplant (such as donation after brain death (DBD) or donation after circulatory death (DCD) transplants). Furthermore, no attempt is made to pair estimated graft life with estimated recipient survival. In several countries, there is now great interest in developing organ allocation schemes based on transplant benefit, while the USA has introduced an allocation procedure taking into account the estimated post-transplant survival and the donor kidney quality (as measured by the kidney donor profile index).^30^

The principles of organ allocation procedures based on net benefit involve the calculation of scores that reflect the potential benefit of transplantation based on comprehensive outcome analyses, an individual's life expectancy with and without a given transplant and to prioritise patients who have most to gain. At a point when a donor organ becomes available, the expected number of days of life without a transplant can be compared with the expected number of days of life following receipt of a transplant. This procedure requires the development of statistical models for survival following wait listing and for survival post-transplantation.

On the basis of the information obtained in the study, we will also explore deceased donor kidney allocation (including kidneys from DCD donors) on the basis of a continuous index of donor organ longevity, along with a continuous index of potential transplant recipients that predicts their likely survival when transplanted over that on dialysis (ie, life years gained due to transplantation). We will incorporate information on QoL into the allocation model by assigning scores for transplantation with different types of organs (ie, DCD or DBD) versus dialysis, informed by the PROMs workstream. Similarly, the cost-effectiveness of transplantation with different types of donor organs could be explored in the model. These data will then be assessed alongside other factors that predict length of wait and survival enabling the development of model(s) which predict an accurate difference in the overall net benefit of a particular type of transplant, thus maximising organ utilisation and the overall benefit for the patients. The impact of potential models of organ allocation will be tested using simulations where the properties of different schemes can be explored and compared, and the impact of policy changes can be forecast. Allocation schemes that focus on different aspects, such as maximum benefit from an organ or equal access to transplantation, can be simulated and the results used to help identify an allocation scheme that provides a balance between efficiency and equity that is acceptable to patients and society.

## Ethics and dissemination

Renal transplantation is one of the most successful therapies in modern medicine. However, the landscape of renal transplantation has changed significantly over the last decade with an increasing need, in an older population with more comorbidities and a different donor population, with a higher number of extended criteria donors and DCD. As a consequence, there are a number of major challenges currently facing the provision of renal transplant services. Some of these challenges raise ethical concerns regarding the transparency of the selection process, the consistency of the decision-making process and the equity of access to the transplantation. These issues are at the core of ATTOM and the involvement of patients and ethicists throughout the design and conduct of the study are key to the success of this programme.

Comorbidity, particularly cardiovascular comorbidity, is common in patients with chronic kidney disease (CKD) and may be an important factor leading to inequity in access to transplantation.^10^ Previous studies have demonstrated that demographic variables such as gender, age, geographical location and level of social deprivation influence access to transplantation^10^ ^14^ ^46–49^ and their interpretation varies significantly between centres, raising further concerns about an equal chance of consideration for transplantation. Unlike previous reports, which are retrospective or based on registry analyses, ATTOM is collecting prospective comorbidity data at the time of patients starting dialysis and assesses its impact according to the outcome as shown in figure 4. Furthermore, the planned analyses will enable us to assess further potential inequities in access to transplantation after listing and establish the impact of comorbidity and sociodemographic variables on the outcome of renal transplantation, SPK transplantation and dialysis. The study design and the data collected in ATTOM allow individual patient predictions to be generated, facilitating more informed decision-making. Importantly, it will provide uniformly applicable and explicit evidence-based assessment criteria for entry onto the national transplant waiting-list for kidney and SPK transplantation addressing some of the major ethical concerns highlighted above.

Combining a quantitative and qualitative analysis is one of the novel aspects of ATTOM, allowing an in-depth analysis of individual centre practices, policies and beliefs as well as the views held by patients. By identifying the recipient and organisational factors that most influence access to transplantation and subsequent transplant outcome, the findings will address key ethical concerns and indicate where clinical practice can be changed or refined to achieve fairer and more transparent access to transplantation.

The impact of comorbidity on SPK transplantation outcomes is also unclear, particularly given the more stringent selection criteria for this procedure.^50^ There is an ongoing debate regarding the survival benefit of SPK transplantation over and above renal transplantation alone, particularly living donor renal transplantation. ATTOM addresses this issue by directly comparing outcomes in patients taking account of differences in sociodemographics and comorbidity.

There is a strong perception that successful kidney transplantation improves health-related QoL compared with dialysis. One of the ATTOM workstreams addresses these issues providing information on quality of health, QoL, well-being and treatment satisfaction using a combination of established generic instruments as well as recently developed condition-specific measurement tools designed for people with CKD. Furthermore, the study may identify which particular subgroups of patients are likely to gain most or least from transplant because of comorbid disease.

ATTOM includes a health economic analysis that provides insight into long-term cost and survival differences associated with dialysis and transplantation. While the effectiveness of transplantation has already been established, ATTOM considers current clinical pathways and enables further exploration of the impact of donor and recipient factors on both costs and outcomes in the modelling of alternative approaches to allocating organs in the UK.

Organ allocation schemes (addressed in workstream 5) and issues such as which patients should receive priority, which organs should be used and which criteria should inform the allocation decision are at the heart of ethical debates in transplantation.

Data from this study will be curated by the NHSBT and UK Registry providing an ethical reassurance regarding the use of the information collected in the study.

The results of ATTOM will be of direct relevance to patients and their clinicians, and are expected to reshape the provision of renal transplantation in the UK by evaluating the entire CKD pathway from dialysis to transplantation. From a public perspective, ATTOM will provide unprecedented transparency in the decision-making with regard to the use of a scarce national resource. Therefore, we plan to disseminate these findings widely in peer-reviewed journals, at national and international conferences and thorough public engagement days. Furthermore, we intend to engage all relevant stakeholders in the discussions concerning any proposed alternative organ allocation schemes.

In conclusion, ATTOM is the first research programme involving all renal dialysis and renal transplant units in the UK that explores in depth the relationship between access to transplantation and transplant outcomes. The outputs of the study are likely to have a significant impact on the delivery of renal transplantation in the UK.

## References

[1] Wolfe RA, Ashby VB, Milford EL Comparison of mortality in all patients on dialysis, patients on dialysis awaiting transplantation and recipients of a first cadaveric transplant. N Engl J Med 1999;341:1725–30. 10.1056/NEJM199912023412303 10580071

[2] Oniscu GC, Brown H, Forsythe JLR Impact of cadaveric renal transplantation on survival in patients listed for transplantation. J Am Soc Nephrol 2005;16:1859–65. 10.1681/ASN.2004121092 15857921

[3] Neipp M, Karavul B, Jackobs S Quality of life in adult transplant recipients more than 15 years after kidney transplantation. Transplantation 2006;81:1640–4. 10.1097/01.tp.0000226070.74443.fb 16794528

[4] Abecassis M, Bartlett ST, Collins AJ Kidney transplantation as primary therapy for end-stage renal disease: a National Kidney Foundation/Kidney Disease Outcomes Quality Initiative (NKF/KDOQITM) conference. Clin J Am Soc Nephrol 2008;3: 471–80. 10.2215/CJN.05021107 18256371 PMC2390948

[5] Tyden G, Bolinder J, Solders G Improved survival in patients with insulin-dependent diabetes mellitus and end-stage diabetic nephropathy 10 years after combined pancreas and kidney transplantation. Transplantation 1999;67:645–8. 10.1097/00007890-199903150-00001 10096516

[6] Sureshkumar KK, Patel BM, Markatos A Quality of life after organ transplantation in type 1 diabetics with end-stage renal disease. Clin Transplant 2006;20:19–25. 10.1111/j.1399-0012.2005.00433.x 16556148

[7] Gaylin DS, Held PJ, Port FK The impact of comorbid and sociodemographic factors on access to renal transplantation. JAMA 1993;269:603–8. 10.1001/jama.1993.03500050081030 8421364

[8] Van Manen JG, Korevaar JC, Dekker FW, NECOSAD-Study Group. Adjustment for comorbidity in studies on health status in ESRD patients: which comorbidity index to use? J Am Soc Nephrol 2003;14:478–85. 10.1097/01.ASN.0000043902.30577.C9 12538750

[9] Rodger S, Forsythe JL, Bradley JA Transplantation. In The Renal Association, ed.: Treatment of adults and children with renal failure. 3rd edn Sudbury, Suffolk: Royal College of Physicians of London. The Lavenham Press Ltd, 2002;95 115.

[10] Oniscu GC, Schalkwijk AA, Johnson RJ Equity of access to renal transplant waiting list and renal transplantation in Scotland: cohort study. BMJ 2003;327:1261 10.1136/bmj.327.7426.1261 14644969 PMC286245

[11] Dudley CR, Johnson RJ, Thomas HL Factors that influence access to the national renal transplant waiting list. Transplantation 2009;88:96–102. 10.1097/TP.0b013e3181aa901a 19584687

[12] Akolekar D, Oniscu GC, Forsythe JL Variations in the assessment practice for renal transplantation across the United Kingdom. Transplantation 2008;85:407–10. 10.1097/TP.0b013e3181629bac 18322433

[13] Ravanan R, Udayaraj U, Ansell D Variations between centres in access to renal transplantation in UK: longitudinal cohort study. BMJ 2010;341:c3451 10.1136/bmj.c3451 20647283 PMC2907479

[14] Kiberd B, Boudreault J, Bhan V Access to the kidney transplant wait list. Am J Transplant 2006;6:2714–20. 10.1111/j.1600-6143.2006.01523.x 16952294

[15] Balaska A, Moustafellos P, Gourgiotis S Changes in health-related quality of life in Greek adult patients 1 year after successful renal transplantation. Exp Clin Transplant 2006;4: 521–4. 17238851

[16] Liem YS, Bosch JL, Arends LR Quality of life assessed with the Medical Outcomes Study Short Form 36-item Health Survey of patients on renal replacement therapy: a systematic review and meta-analysis. Value Health 2007;10:390–7. 10.1111/j.1524-4733.2007.00193.x 17888104

[17] Maglakelidze N, Pantsulaia T, Tchokhonelidze I Assessment of health-related quality of life in renal transplant recipients and dialysis patients. Transplant Proceedings 2011;43:376–9. 10.1016/j.transproceed.2010.12.015 21335226

[18] Sayin A, Mutluay R, Sindel S Quality of life in hemodialysis, peritoneal dialysis, and transplantation patients. Transplant Proc 2007;39:3047–53. 10.1016/j.transproceed.2007.09.030 18089319

[19] Bradley C Importance of differentiating health status from quality of life. Lancet 2001;357:7–8. 10.1016/S0140-6736(00)03562-5 11197385

[20] Laupacis A, Keown P, Pus N A study of the quality of life and cost-utility of renal transplantation. Kidney Int 1996;50:235–42. 10.1038/ki.1996.307 8807593

[21] Howard K, Salkeld G, White S The cost-effectiveness of increasing kidney transplantation and home-based dialysis. Nephrology (Carlton) 2009;14:123–32. 10.1111/j.1440-1797.2008.01073.x 19207859

[22] Merion RM, Schaubel DE, Dykstra DM The survival benefit of liver transplantation. Am J Transplant 2005;5:307–13. 10.1111/j.1600-6143.2004.00703.x 15643990

[23] Egan TM, Murray S, Bustami RT Development of the new lung allocation system in the United States. Am J Transplant 2006;6:1212–27. 10.1111/j.1600-6143.2006.01276.x 16613597

[24] Audard V, Matignon M, Dahan K Renal transplantation from extended criteria cadaveric donors: problems and perspectives overview. Transpl Int 2008;21:11–17. 10.1111/j.1432-2277.2007.00543.x 17850235

[25] Salvalaggio PR, Schnitzler MA, Abbott KC Patient and graft survival implications of simultaneous pancreas kidney transplantation from old donors. Am J Transplant 2007;7: 1561–71. 10.1111/j.1600-6143.2007.01818.x 17511681

[26] Locke JE, Segev DL, Warren DS Outcomes of kidneys from donors after cardiac death: implications for allocation and preservation. Am J Transplant 2007;7:1797–807. 10.1111/j.1600-6143.2007.01852.x 17524076

[27] Dahmane D, Audard V, Hiesse C Retrospective follow-up of transplantation of kidneys from ‘marginal’ donors. Kidney Int 2006;69:546–52. 10.1038/sj.ki.5000102 16407884

[28] Saidi RF, Elias N, Kawai T Outcome of kidney transplantation using expanded criteria donors and donation after cardiac death kidneys: realities and costs. Am J Transplant 2007;7:2769–74. 10.1111/j.1600-6143.2007.01993.x 17927805

[29] Oniscu GC, Brown H, Forsythe JLR Validation of a mortality risk-prediction model as a basis for listing patients on the kidney transplant waiting list. Am J Transplant 2005;5 (S11):403.

[30] http://optn.transplant.hrsa.gov/PublicComment/pubcommentPropSub_311.pdf (accessed on 8 May 2013).

[31] Herdman M, Gudex C, Lloyd A Development and preliminary testing of the new five-level version of EQ-5D (EQ-5D-5L). Qual Life Res 2011;10:1727–36. 10.1007/s11136-011-9903-x PMC3220807 21479777

[32] Riazi A, Bradley C, Barendse S Development of the Well-being questionnaire short-form in Japanese: the W-BQ12. Health Qual Life Outcomes 2006;4:40 10.1186/1477-7525-4-40 16817960 PMC1563454

[33] Bradley C The 12-item Well-Being Questionnaire. Origins, current stage of development, and availability. Diabetes Care 2000;23:875 10.2337/diacare.23.6.875 10841025

[34] McMillan CV, Bradley C, Gibney J Psychometric properties of two measures of psychological well-being in adult growth hormone deficiency. Health Qual Life Outcomes 2006;4:16 10.1186/1477-7525-4-16 16553952 PMC1475840

[35] Mitchell J, Bradley C Psychometric evaluation of the 12-item Well-being Questionnaire for use with people with macular disease. Qual Life Res 2001;10:465–73. 10.1023/A:1012540100613 11763208

[36] Bradley C Design of a renal-dependent individualised quality of life questionnaire: RDQoL. Adv Perit Dial 1997;13:116–20. 9360663

[37] Bradley C, Todd C, Gorton T The development of an individualised questionnaire measure of perceived impact of diabetes on quality of life: the ADDQoL. Qual Life Res 1999;8:79–91. 10.1023/A:1026485130100 10457741

[38] Wee HL, Tan CE, Goh SY Usefulness of the Audit of Diabetes Dependent Quality of Life (ADDQoL) questionnaire in patients with diabetes in a multi-ethnic Asian country. Pharmacoeconomics 2006;24:673–82. 10.2165/00019053-200624070-00006 16802843

[39] Barendse SM, Speight J, Bradley C The Renal Treatment Satisfaction Questionnaire (RTSQ): a measure of satisfaction with treatment for chronic kidney failure. Am J Kidney Dis 2005;45:572–9. 10.1053/j.ajkd.2004.11.010 15754280

[40] Bradley C The Diabetes Treatment Satisfaction Questionnaire: DTSQ. In: Bradley C, ed. Handbook of psychology and diabetes: a guide to psychological measurement in diabetes research and practice. Chur, Switzerland: Harwood Academic Publishers, 1994:111–32.

[41] Bradley C, Lewis KS Measures of psychological well-being and treatment satisfaction developed from the responses of people with tablet-treated diabetes. Diabet Med 1990;7:445–51. 10.1111/j.1464-5491.1990.tb01421.x 2142043

[42] Howorka K, Pumprla J, Schlusche C Dealing with ceiling baseline treatment satisfaction level in patients with diabetes under flexible, functional insulin treatment: assessment of improvements in treatment satisfaction with a new insulin analogue. Qual Life Res 2000;9:915–30. 10.1023/A:1008921419108 11284211

[43] Bradley C The Diabetes Treatment Satisfaction Questionnaire (DTSQ): change version for use alongside status version provides appropriate solution where ceiling effects occur. Diabetes Care 1999;22:530–2. 10.2337/diacare.22.3.530 10097946

[44] Bradley C, Plowright R, Stewart J The Diabetes Treatment Satisfaction Questionnaire change version (DTSQc) evaluated in insulin glargine trials shows greater responsiveness to improvements than the original DTSQ. Health Qual Life Outcomes 2007;5:57 10.1186/1477-7525-5-57 17927832 PMC2170436

[45] Karnon J, Stahl J, Brennan A Modeling using discrete event simulation: a report of the ISPOR-SMDM Modeling Good Research Practices Task Force-4. Med Decis Making 2012;32:701–11. 10.1177/0272989X12455462 22990085

[46] Wolfe RA, Ashby VB, Milford EL Differences in access to cadaveric renal transplantation in the United States. Am J Kidney Dis 2000;36:1025–33. 10.1053/ajkd.2000.19106 11054361

[47] Miceli M, Di Lallo D, Perucci CA Absence of economic barriers does not reduce disparities in the access to renal transplantation: a population based study in a region of central Italy. Dialysis Register of Lazio Region. J Epidemiol Community Health 2000;54:157–8. 10.1136/jech.54.2.157 10715750 PMC1731627

[48] Schaubel DE, Stewart DE, Morrison HI Sex inequality in kidney transplantation rates. Arch Intern Med 2000;160:2349–54. 10.1001/archinte.160.15.2349 10927733

[49] Jindal RM, Ryan JJ, Sajjad I Kidney transplantation and gender disparity. Am J Nephrol 2005;25:474–83. 10.1159/000087920 16127268

[50] Vanrenterghem YF, Claes K, Montagnino G Risk factors for cardiovascular events after successful renal transplantation. Transplantation 2008;85:209–16. 10.1097/TP.0b013e318160254f 18212625

