# Supplementary material for: Access to Transplantation and Transplant Outcome Measures (ATTOM): study protocol of a UK wide, in-depth, prospective cohort analysis
Source: BMJ Open. 2016 Feb 25;6(2):e010377. doi: 10.1136/bmjopen-2015-010377 (PMC4769394; doi:10.1136/bmjopen-2015-010377)
Supplement: Supplementary data [file bmjopen-2015-010377supp.pdf]

ATTOM data sheet

DEMOGRAPHICS

Patient group:
Choose one of 3 options. Groups self explanatory

Cohort:
Choose from one of 2 options. ‘Detailed PROMs cohort’ will only be relevant to patients in certain centres + centres undertaking pancreas transplantation

Name/DOB/Sex:
Self explanatory. System limit – no later than 31/12/1994, no earlier than 01/01/1935

Ethnicity:
Choose one of 5 options.
White – patient appears or is recorded as being of white ethnicity
Black – patient appears black or is recorded as being of black ethnicity
Mixed – patient appears or is recorded as being of mixed (any combination) of ethnic parentage
Asian – patient appears or is recorded as being of Indian/Pakistani/Bangladeshi ethnicity
Chinese – patient appears or is recorded as being of Chinese ethnicity

Height:
Height in centimeters – system limit of 100 – 240 cms

Weight:
Weight in kilograms – dry weight/target weight as far as possible
System limit 30-220 kgs
Decimal places not allowed

Patient email id:
Entry only required if patient requests e-access for completing questionnaires. Entering email id will generate an auto-email to patient with a password to enable access to the web site

Centre:
Transplant centre where nurse is employed. Not related to centre caring for patient

Renal unit:
Name of renal unit (transplanting or non-transplanting) to which patient belongs

Hospital number:
Unique id number (with or without alphabets) in the hospital with primary care for patient (hospital that undertakes the patient’s dialysis treatment)
Other number – unique id number in secondary hospital (for ex: transplant centre) where the patient may have had tertiary care

NHS number (for patients in England, Wales and Northern Ireland only):
Invalid NHS numbers will not be accepted / saved (internal modulus 11 algorithm check)

CHI number (for patients in Scotland only)
Invalid CHI number will not be accepted / saved

Address/Post code:
Address as listed in the IT system of the hospital that provides primary care for the patient.

Date first seen by Nephrologist:
Date when first seen by Nephrologist (either in clinic or as an in-patient)
If information on all 3 variables day/month/year – enter exact date
If information on only month/year available – enter 15th of that month
If information on only year available – enter 30th June for that year

Date of data entry:
Date when you started filling in the demographics page for this patient for the first time (may or may not be the same date as obtaining consent from the patient for study participation)

Mandatory data items for the demographics page:
Patient group
Cohort
First name
Surname
DOB
Address & Post code

SOCIOECONOMIC DATA

(cannot save data on this page without entering details on demographic page first)

Language

Is English your first language? Self explanatory yes/no. If no chosen - pop up of 'what is your first language?' self explanatory – free text box for writing language
Pop up box of 'Please rate your fluency in English?' – ask patient to choose from 'basic/moderate/good fluency' as reported by the patient

Leading question ("How often do you need ….….") on help required for medical instructions – give patients the 5 choices and record patient preference

Place of birth
Self explanatory question. No follow up questions

Ethnicity
Record patient reported preferences from choices listed. If 'other' is chosen from any category – pop up free text box

Education
Record patient reported educational qualifications. More than 1 and a maximum of 5 options can be ticked. Highest achieved qualification must be selected.

Employment status
Record patient preference from list of 8 options. Patient must report status from the preceding 4 weeks

Car ownership
Self explanatory question. Only 3 or 4 wheeled motorised vehicles to be counted. If ‘yes’ ticked pop up question of ‘How many vehicles’. No system limit to number of vehicles

Housing
Record patient preference for self explanatory question. If ‘other’ chosen pop up free text box

Civil status
Record patient preference for self explanatory question.

Dependants
Question implies number of people living in the same household and not necessarily dependant in financial or social terms. Children classified as <18years and adults as aged 18 or over. No system limits to number entered

Smoking
Record patient preference for self explanatory question. If ‘yes’ is ticked pop up question on number of cigarettes. If ‘no’ is ticked pop up question on previous smoking habit. If ‘yes’ ticked for previous smoker pop up question on duration since last smoked (aim to get the closest number of years unless it is <12 months since smoking cessation)

COMORBIDITY

(cannot save data on this page without entering details on demographic page first)

For the following data items, please read case notes (admission clerking notes, inter-specialty referral letters, discharge summaries are particularly useful), clinic letters, local renal IT systems or based on reports from patient/patient’s named consultant. Record data items as per information gathered from above sources. If in doubt for any item check with the patient’s named consultant nephrologist. For each item – if both month and year known, enter exact month/year. If only year known enter June as default month. Exact date of diagnosis for long term conditions (for ex: Diabetes, Asthma/COPD etc) is difficult to ascertain and in such situations ask patient how many years they have had this diagnosis and then choose that year in ‘year’ box and then choose default options of ‘15th’ and ‘June’ for day and month respectively.
For each data item – if more than one entry is needed, please click on the ‘add’ button to the right of the ‘Month/Year’ tabs to open a new box. Up to a maximum of 3 boxes can be opened for each data item.

Primary Renal diagnosis

This data item indicates cause of kidney failure. Usually specified in clinic letters, local renal IT systems, patient reported cause etc. If 'other' is chosen, please fill pop up free text box Code numbers help link presumed diagnosis to registry records.

Diabetes

This data item indicates whether the patient has diabetes or not (irrespective of whether the diabetes caused kidney failure or not)

If 'yes' is checked pop up box of type I or II

Type I – diagnosis must be before age 30 years, must be on Insulin from day 1 of diabetes, may have had previous episodes of diabetic ketoacidosis. Type II – diagnosed after the age of 30, may have had diet/tablets/insulin as treatment for diabetes. This includes diabetes induced by drugs such as Ciclosporin/Tacrolimus, patients who developed diabetes after pancreatectomy/pancreatitis etc.

Ischaemic heart disease

This data item indicates whether the patient suffers from / has suffered from Ischaemic/coronary heart disease.

Angina – diagnosis of angina as recorded in case notes or reported by patient. Usually implies typical sounding cardiac chest pain, often on exertion, relieved by GTN/rest etc.

NSTEMI – diagnosis of non-ST segment elevation MI or acute coronary syndrome without ECG changes (i.e. raised troponin levels). Can only be diagnosed following blood test + ECG and therefore cannot be reported as an event by patient

STEMI/MI – diagnosis of ST elevation MI with obvious ECG changes. Can only be diagnosed with an ECG

Coronary intervention – patient has had an intervention for presumed ischaemic heart disease (with or without previous history of angina/NSTEMI/STEMI). Please choose between PCI (coronary angioplasty with or without stent insertion) or CABG (bypass operation)

Heart failure

This data item indicates whether the patient suffers from heart failure. Indicate 'yes' if any of the following items appear to have been diagnosed according to the case notes/clinic letters

Congestive cardiac failure or CCF

Left ventricular failure or LVF

Right ventricular failure of RVF

LV or RV dysfunction on ECHO

Ejection fraction or EF <30% on ECHO

Atrial Fibrillation

This data item indicates whether the patient is in atrial fibrillation currently. Do not choose 'yes' if patient had previous episodes of atrial fibrillation but is not in AF currently.

Cardiac valve replacement

This data item indicates whether the patient had a previous cardiac valve replacement or valve repair surgery. If 'yes' ticked, pop up box of which valve was replaced/repaired and month/year of procedure.

Permanent pacemaker
This data item indicates whether the patient currently has a permanent pacemaker in-situ. If ‘yes’ is ticked, pop up box of month/year of insertion
Cerebrovascular disease
This data item indicates whether the patient has had symptomatic cerebrovascular disease or cerebrovascular intervention. If ‘yes’ is ticked pop up box of type of event.
TIA – Indicate if TIA(transient ischaemic accident) /mini-stroke/transient stroke appears in case notes/letters
CVE/Stroke – Indicate if CVE or CVA (cerebro-vascular event or accident) /Stroke/hemiplegia/cerebral haemorrhage/sub-arachnoid haemorrhage/sub-dural haemorrhage appears in case notes/letters
Carotid intervention – indicate if carotid endartrectomy or carotid angioplasty or carotid operation appears in case notes

Peripheral vascular disease
This data item indicates whether the patient suffers from peripheral (usually lower limb) vascular disease. If ‘yes’ is ticked pop up box of type of event.
Claudication – indicate if claudication (lower limb pain on walking) appears in case notes
Radiological or surgical intervention – indicate if iliac or femoral or popliteal or profunda or anterior tibial or posterior tibial artery intervention (angioplasty, endartrectomy, bypass etc) appears in case notes
Amputation – indicate if any amputation of any part of any limb (except traumatic amputation or penile amputation) appears in case notes

Abdominal Aortic Aneurysm
This data item indicates whether the patient has ever been diagnosed as having or treated for a AAA. If AAA is indicated any where in case notes tick ‘yes’ and specify whether the AAA is just being monitored or whether radiological (EVAR) or open surgical procedure (AAA repair) has been undertaken.

Respiratory disease
This data item indicates whether the patient suffers from any form of respiratory disease. If any of the terms including ‘Asthma’, ‘COPD’, ‘Emphysema’ or ‘Bronchiectasis’ appears in the case notes tick ‘yes’ and specify which/how many of the 3 diagnoses is relevant to the patient. Emphysema can be coded as COPD.
Liver Disease
This data item indicates whether the patient suffers from any form of liver disease. If the term ‘Cirrhosis’, ‘Non Alcoholic steato-hepatitis or NASH’, ‘Drug induced (for ex: paracetamol poisoning’ liver disease and ‘Alcoholic liver disease’ appears in the case notes tick ‘yes’. If the word cirrhosis is used in the case notes choose ‘cirrhotic liver disease’ from the drop down menu. If liver disease is mentioned without the term cirrhosis then choose ‘non-cirrhotic liver disease’.
Note – cholecystitis / gall stones etc does not constitute liver disease

Blood Borne Viruses
This data item indicates whether the patient suffers/has suffered from BBV infection. If Hepatitis C/B/HIV infection (past or present) or Hep C/B PCR or antibody positive

or HIV PCR/antibody positive is recorded in case notes tick ‘yes’ and then indicate which/how many viral infections is relevant to the patient.

Malignancy

This data item indicates whether the patient has been diagnosed with one or more malignancies in the past. If any malignancy has been recorded in the case notes, tick ‘yes’ and then specify which type of malignancy from the drop down menu. Please note – tick ‘yes’ only for a malignancy. Benign tumours (such as breast adenoma, colon polyp, skin warts/actinic keratosis etc do not count as malignancy).

Mental illness

This data item indicates whether the patient has suffered from/suffers from any recorded mental illness in the case notes. Tick ‘yes’ if the term ‘Depression’, ‘Psychosis/Psychotic disorder’, ‘Bipolar disorder’, ‘substance abuse’ (usually indicates poisoning with one or more drugs – not alcohol or recreational drugs) and ‘deliberate self harm’ (usually indicates physical attempts at self harm – not chemical means which should be classified under ‘substance abuse’) or related terms such as ‘Schizophrenia’ (should be classified as a psychotic disorder) appears in the case notes. If in doubt ask the patient / consultant nephrologist/ named psychiatric nurse.

Dementia

This data item indicates whether the patient suffers from any form of dementia. Tick ‘yes’ if the term ‘dementia’, ‘vascular dementia’, ‘Alzheimer’s disease’, ‘memory loss’ (short or long term) etc appears in the case notes. If in doubt, please check with the consultant Nephrologist.

Smoking

This data item captures whether the patient’s smoking history is available in the case notes and therefore fill in the data item purely based on information available in the case notes. This may or may not contradict what the patient reports in the socio-economic questionnaire. If the term ‘smoker’, ‘heavy smoker’ etc is recorded in case notes indicate patient is a current smoker. If the case notes indicate that patient is an ‘ex-smoker’ , ‘quit xx years ago’ etc indicate patient is an ex-smoker. If the notes indicate that the patient has never smoked indicate ‘non-smoker’. If there is no mention of smoking anywhere in the notes – indicate ‘don’t know’.

Other illness

3 x free text boxes to indicate any other illness that does not come under the above topic headings.

Incident dialysis

(cannot save data on this page without entering details on demographic page first)
(Demographic, comorbidity and socio-economic data same as for all patients)

<u>Start date of dialysis</u>

Indicated when the patient commenced long term / permanent dialysis treatment. For many patients it will be planned start on dialysis (for ex: after PD catheter insertion or AVF formation). This should be the date of the first ever dialysis session (PD or HD) even if the patient subsequently changed modalities. If the patient started dialysis as an ‘acute patient’ recovered renal function for a little while and then re-started dialysis, indicate date when dialysis was re-started. For patient crash landing on dialysis treatment (starting dialysis without prior planning – usually during an in-patient admission) record date of first dialysis session (usually HD session and rarely PD) as date of first dialysis. If in doubt check with local renal IT system or ask consultant nephrologist / dialysis unit sister.

<u>Type of dialysis</u>

This should be the dialysis modality that the patient started on when dialysis first commenced. The type of dialysis modality is self explanatory and if not clear please check with the HD unit sister to confirm between HD-v-HDF and PD unit sister between APD-v-CAPD.

If patient has been on more than one type of modality between start of treatment and time of consenting to participate in this research project, tick the modality that the patient has spent most time on.

If HD or HDF is chosen, pop up menu of type of dialysis access. This indicates the type of HD access at the start of HD/HDF (first ever HD/HDF session). The types of access are self explanatory. Non-tunnelled lines are also often referred to as ‘Vascath’ and tunnelled lines are often referred to as ‘Permcath’ or ‘Tesio’.

If the patient has used more than one type of access between start of HD/HDF and time of consenting the patient for participation in the research project, tick the access type that was most used since starting HD/HDF. If patients were using one needle in AVF/AVG and one needle in tunnelled line/non-tunnelled line – tick tunnelled line/non-tunnelled line as access used.

<u>Previous transplant</u>

This data item indicates whether the patient has had a previous organ transplant (any solid organ and not just kidney only). The previous organ transplant may or may not be still working (for ex: working liver transplant as compared to failed kidney transplant). Indicate number of previous transplants and then indicate type of organ transplant. If exact day/month/year of previous transplant known, enter exact date. If only month/year known – enter 15th of the month/year. If only year known – enter 30th June of the year.

Incident transplant patient
Transplant work up

(cannot save data on this page without entering details on demographic page first)
(Demographic, comorbidity and socio-economic data same as for all patients)

Cardiac
Indicates whether the patient had any cardiac investigations were undertaken as part of the work up / declaration of fitness for kidney (or kidney + pancreas transplant). The investigations undertaken are likely to be listed in the case notes or described in clinic letters (either from the nephrologist or cardiologist). The result of the test is not relevant to this data item but just whether any tests were done or not done. Please include only tests done as part of work up for transplantation (usually done prior to transplant wait-listing or prior to transplant) and not include tests done in the past. If no tests are apparent then please tick 'none'.
More than one option can be ticked.

Pulmonary
Indicates whether the patient had any pulmonary function tests (includes lung function tests and CPEX or cardio-pulmonary exercise testing). If no tests are apparent then please tick 'none'.

Vascular
Indicates whether the patient had any vascular investigations (iliac/lower limb and carotid only – upper limb vascular investigations should not be included). Clinic letters from the vascular surgeons / transplant surgeons are likely to be the best sources of information. If no tests are apparent, then please click 'none'. More than one option can be ticked.

Other tests
If any other tests (for ex: genetic tests, CT/MRI scans of other organs, other radiological tests, blood tests such as Glucose Tolerance Tests etc) were undertaken exclusively for the purpose of confirming fitness for transplantation, please list then the free text box. Up to 3 items can be entered.

Incident transplant information

Date of transplant
Indicates date of renal (or renal + pancreas) transplant that triggered entry into the ATTOM study. Please enter exact date of transplant.

Transplanted organ
Indicates whether this was a kidney only or kidney + other organ transplant.

Transplant type
Indicates whether the organ/s came from a live donor or brain dead (DBD/HBD) or non-heart beating (DCD/NHBD) donor.

Treatment modality
Indicates what form of dialysis if any the patient was having just before the transplant.

If any modality (other than preemptive or failing transplant) is chosen please fill in date when the patient started dialysis for the first time. This helps calculate total time on dialysis before transplant. If HD/HDF was chosen, please also complete additional pop up menu of type of dialysis access.

Patient has had a previous transplant?

This data item indicates whether the patient has had any previous organ transplant (not just kidney). Please indicate type of transplant and date (using default options of 15th for the day and June for the month if either not known).

Induction immune suppression

Indicates the type of drug given just before the transplant operation. This is usually an IV drug and the common drugs used are listed. If ‘other’ is chosen – please fill in name of drug in the free text box

Maintenance CNI

CNI stands for ‘Calcineurin inhibitor’ and can only be Ciclosporin or Tacrolimus. Please indicate ‘Tacrolimus’ or ‘ciclosporin’ irrespective of whether the primary brand or generic brand of the drug is used. Maintenance therapy usually indicates that the patient is likely to remain on this drug for the foreseeable future.

Maintenance anti-proliferative

This data item captures whether the patient is likely to continue on any anti-proliferative agent for the foreseeable future. This is usually an oral medication and the common drugs are listed. Please pick from the drop down menu.

Maintenance steroid

This data item captures whether the patient is likely to be on short or long term steroid (usually Prednisolone) treatment. If unit policy is that for all/most patients to be weaned off steroids at 1 or 3 months, then please tick this option. If unit policy is for steroid continuation for >3 months but not indefinitely (say 6 or 12 months) please tick ‘long term continuation’.

Maintenance other

This data item captures whether the patient is on any other long term immune suppression using drugs not in any of the above categories. This maybe a oral drug or IV/SC drug (Belatacept) and the common drugs are listed. Please tick any appropriate choice.

Matched control for transplant patient

(cannot save data on this page without entering details on demographic page first)
(Demographic, socio-economic info and comorbidity same as for all patients)
Transplant work up information – same as above

Wait-listing information

Date of activation on the waiting list
Please indicate the date of very first activation on the waiting list (irrespective of any subsequent suspensions etc). Data should normally be available with the transplant coordinators, renal IT system and less rarely in the notes.

Organ
The data item captures which organ/s the patient was first listed for. If the patient was listed for a kidney only and subsequently listed for a kidney + other organ – please tick ‘kidney only’ as this was the choice at time of first listing.

Dialysis modality at time of data collection
Data item captures type of dialysis at the time the patient was recruited to the ATTOM study. If HD/HDF chosen, please indicate type of access. Please leave blank if patient is currently not on any form of dialysis (preemptively listed or has a failing transplant but not yet back on dialysis)
Please do not fill in ‘supervising hospital’. This data field is not required.

Patient had a previous transplant
This data item captures if the patient has had a previous organ (not kidney only) transplant. Please indicate exact date of transplant if known, and if not use default options of 15th if day not known and 30th June if month not known.
